# Sugar Beet Molasses as a Potential C-Substrate for PHA Production by *Cupriavidus necator*

**DOI:** 10.3390/bioengineering9040154

**Published:** 2022-04-04

**Authors:** Evgeniy G. Kiselev, Aleksey V. Demidenko, Natalia O. Zhila, Ekaterina I. Shishatskaya, Tatiana G. Volova

**Affiliations:** 1 Basic Department of Biotechnology, School of Fundamental Biology and Biotechnology, Siberian Federal University, 79 Svobodnyi Av., 660041 Krasnoyarsk, Russia; evgeniygek@gmail.com (E.G.K.); kraysolnca@mail.ru (A.V.D.); shishatskaya@inbox.ru (E.I.S.); volova45@mail.ru (T.G.V.); 2 Institute of Biophysics SB RAS, Federal Research Center “Krasnoyarsk Science Center SB RAS”, 50/50 Akademgorodok, 660036 Krasnoyarsk, Russia

**Keywords:** sugar beet molasses, hydrolysis, *Cupriavidus necator*, synthesis, properties of PHA

## Abstract

To increase the availability and expand the raw material base, the production of polyhydroxyalkanoates (PHA) by the wild strain *Cupriavidus necator* B-10646 on hydrolysates of sugar beet molasses was studied. The hydrolysis of molasses was carried out using *β*-fructofuranosidase, which provides a high conversion of sucrose (88.9%) to hexoses. We showed the necessity to adjust the chemical composition of molasses hydrolysate to balance with the physiological needs of *C. necator* B-10646 and reduce excess sugars and nitrogen and eliminate phosphorus deficiency. The modes of cultivation of bacteria on diluted hydrolyzed molasses with the controlled feeding of phosphorus and glucose were implemented. Depending on the ratio of sugars introduced into the bacterial culture due to the molasses hydrolysate and glucose additions, the bacterial biomass concentration was obtained from 20–25 to 80–85 g/L with a polymer content up to 80%. The hydrolysates of molasses containing trace amounts of propionate and valerate were used to synthesize a P(3HB-*co*-3HV) copolymer with minor inclusions of 3-hydroxyvlaerate monomers. The introduction of precursors into the medium ensured the synthesis of copolymers with reduced values of the degree of crystallinity, containing, in addition to 3HB, monomers 3HB, 4HB, or 3HHx in an amount of 12–16 mol.%.

## 1. Introduction

The output of indestructible synthetic plastics, which are widely used in all spheres of human activity and obtained from non-renewable resources, has exceeded 380 million tons per year—and their production volumes are constantly growing. The bulk of plastic waste accumulates in landfills, creating a global environmental problem [1,2,3], and causing large-scale environmental pollution, structural damage, and instability in natural ecosystems and threatens beneficial biota and human health [4,5]. Currently, the global average for recycling of plastic waste is about 15%. In case the increase in plastic production and the low level of plastic recycling continues at its current levels, then, by 2050, approximately 12,000 Mt of plastic waste are believed to be in landfills or the natural environment [4]. Studies of the microbiological transformation of synthetic plastics in the natural environment under anaerobic soil conditions and in the waters of the World Ocean [6] have shown that these are extremely long processes. The low efficiency of biodegradation of plastics and the resulting microplastics represent a new and more dangerous environmental threat.

In addition to the prohibitive measures currently in force in more than 60 countries and aimed at reducing the consumption of plastics, the way out is seen in a gradual transition/conversion to new materials that can degrade in the environment without the formation of toxic products. Today, the development of new polymeric materials that can be included in biospheric cycles is one of the high-ranking areas of critical technologies of the 21st century and actualizes research aimed at finding and developing biodegradable “green” plastics as an alternative to synthetic materials [7,8].

Polymers of hydroxyalkanoic acids, polyhydroxyalkanoates (PHAs), are the true products of biotechnology—the so-called “green” plastics. This is a family of polymers with different chemical compositions, different basic properties, and are obtained from various carbon substrates, including waste [9,10,11,12,13,14,15,16]. Such properties as resistance to UV rays, absence of hydrolysis in liquid media, and thermoplasticity make it possible to process PHA into specialized products using available methods from various phase states (solutions, emulsions, powders, and melts) [17,18]. These useful properties, combined with biodegradability and high biocompatibility, put PHA into the category of promising materials for the 21st century and allow them to be considered as a competitor to well-known biodegradable plastics (polylactide, polyethylene terephthalate, polyamides, etc.) for use in various fields from a municipal and agricultural economy to pharmacology and biomedicine [15,16,19,20,21,22].

Potentially, substrates with different degrees of reduction, energy content, and cost can serve as raw materials for PHA production. Among them are individual compounds, as well as complex substrates, including waste [23,24]. The list of microorganisms capable of accumulating PHA includes over 300 bacteria. These are both wild and genetically modified strains.

Promising PHA producers include hydrogen-oxidizing bacteria of the genus *Cupriavidus* (former systematic names: *Wautersia*, *Ralstonia*, *Alcaligenes*, *Hydrogenomonas*) [25], which, in addition to autotrophy and PHA synthesis on mixtures of carbon dioxide and hydrogen [26,27,28,29,30,31], are characterized by a wide organotrophic potential and the ability to synthesize PHA of various chemical structures using various substrates [32,33,34]. However, from the spectrum of sugars, the utilization of which in these microorganisms is realized by the Entner–Doudoroff pathway, they are capable of growing only on fructose, and they are easily susceptible to mutations with the formation of glucose-assimilating mutant strains [35]. Representatives of the *Cupriavidus* taxon are characterized by a high PHA content, which is associated with a powerful intracellular system for the synthesis of reserve polyhydroxyalkanoates and features of growth physiology. In cultures of these microorganisms, even on a complete medium in the middle of the linear growth phase, protein synthesis is inhibited and the formation of poly-3-hydroxybutyrate [P(3HB)] is activated [26,27].

Despite the urgent need for degradable polymeric materials in practice and the high attractiveness of PHA, the increase in their production volumes and the expansion of their applications are constrained by high costs and technical difficulties. Professor G. Chen, the scientific director of one of the world’s leading teams in the field of PHA biotechnology, identifies the following ones:

- the energy intensity of the fermentation process implemented in a strictly sterile batch culture;
- complexity and cost of extraction processes;
- the high crystallinity of the most available PHA poly(3-hydroxybutyrate);
- the difficulty of obtaining more technologically advanced copolymeric PHAs;
- the high cost (up to 40–45%) of the carbon substrate [36].

The key problem of PHA biotechnology is the optimization of the processes of biotechnological synthesis in general, primarily through using new productive strains capable of growing on available substrates and synthesizing PHA of various chemical compositions.

Sugars are the most widely used substrate in biotechnology. Sugar-containing industrial and agricultural wastes, as well as hydrolysates of plant raw materials of various origins, are an inexhaustible renewable substrate resource for the production of target biotechnology products, including PHA [37,38]. The research aimed at the more efficient use of agro-industrial waste for the production of a number of valuable products is being updated. Hydrolysates of various origins have been studied as potential sugar-containing substrates for PHA production: wood [39], sunflower stems [40], miscanthus biomass [41], palm oil [42], cereal straw and husks [43,44,45], sugar beet and sugar cane cakes and peels [46], and others. The research results indicate the promise of plant hydrolysates for productive processes of PHA biosynthesis. However, as a rule, hydrolysates, in addition to hexoses (glucose and fructose) available for PHA producers, also include difficult-to-metabolize sugars, such as galactose and mannose (hexose hydrolysis products), arabinose, and xylose (pentose hydrolysis products). Therefore, to increase the completeness of sugar utilization by microbial cultures during growth on hydrolysates, genetically modified strains with an extended metabolic potential are constructed. For example, in [40], a recombinant *R. eutropha* strain NCIMB11599 (pKM212-XylAB) is described to be capable of utilizing xylose; the *C. necator* W50 strain, which expresses genes from *E. coli*, metabolizes arabinose [47].

Sugar production wastes (cane and beet molasses) are of great importance for biotechnology as a C-substrate [48]. It is an inexpensive carbon source containing, in addition to sugars, vitamins and a spectrum of mineral elements. The main sugar in molasses is disaccharide sucrose, and not all PHA producers have glycosyl hydrolases for the metabolism of sucrose and its transformation into compounds available to cells. Therefore, molasses must be further processed before use. As a result of processing, sucrose hydrolysis occurs and the content of the components that make up the original molasses changes [49]. In addition to glucose and fructose, depending on the hydrolysis method (acidic, alkaline, enzymatic) and conditions (hard or soft), toxic impurities can form in molasses, which negatively affects the biosynthesis process [50]. As a result of the very high content of nitrogen compounds in molasses, which negatively affects its accumulation in PHA cells, it is necessary to adjust the nitrogen concentration as well as the number of mineral elements [51,52].

For the synthesis of PHA using molasses as a C-substrate, two approaches are applicable. The first one is the preliminary hydrolysis of molasses and the sucrose contained in it, and/or the second one is the genetic engineering of recombinant strains capable of metabolizing sucrose. For example, it was shown that molasses can be used as the sole carbon source for the *Klebsiella aerogenes* strain carrying genes for PHB synthesis from *Alcaligenes eutrophus* [53]. In another paper, a recombinant strain of *C. necator* was obtained containing sucrose utilization (csc) genes from *Escherichia coli W*, capable of producing high concentrations of bacterial biomass (up to 113 g/L) with a content of the copolymer P(3HB-*co*-3HHx) up to 70–80% [54]. Additionally, the growth on untreated molasses of another genetically engineered producer, the *Cupriavidus necator* 2058/pCB113 of P(3HB-*co*-3HHx) copolymers, was described [55]. A recombinant strain of *Ralstonia eutropha* is known to contain the sacC gene from *Mannheimias ucciniciproducens*, encoding *β*-fructofuranosidase, which is capable of hydrolyzing sucrose into glucose and fructose and synthesizing a copolymer of poly(3-hydroxybutyrate-*co*-lactate) [P(3HB-*co*-LA)] [56].

Another solution to the problem is to pretreat molasses to hydrolyze sucrose to its glucose and fructose monomers. Sucrose hydrolysis can be carried out using chemical (acid, alkali) or enzymatic (sucrose, invertase) methods [57,58]. It has been shown that the mode of molasses hydrolysis affects the ratio of formed glucose and fructose [57].

The available literature contains a limited number of publications on the study of PHA production on molasses derivatives by representatives of *Cupriavidus* (*Ralstonia*). In one of the first works of this plan, in a culture of *Cupriavidus necator* DSM 545 (formerly known as *Ralstonia eutropha* or *Alcaligenes eutrophus*), it was shown that a small addition of non-hydrolyzed sugar cane molasses (3 g/L) to a medium containing glucose as the main carbon source provides a biomass concentration and polymer content of up to 23 g/L and 39%, respectively [59]. The growth processes of the *Ralstonia eutropha* PTCC1615 bacterium on acid hydrolysates of sugar cane, beet, and soybean molasses were studied [60]. The hydrolysates of sugar cane molasses obtained by various methods, including alkaline or acid hydrolysis—or acid hydrolysis with the preliminary hydrothermal treatment of molasses with oven or oil bath—ensured a polymer accumulation of up to 27% in a *C. nector* culture [57]. In the *C. necator* DSM culture, using pre-hydrolyzed sugar cane molasses with sulfuric acid at high temperatures, the polymer content did not exceed 13% [61]. Vinasse and sugar cane molasses hydrolyzed with acids (HCl or H_2_SO_4_) or with the enzyme invertase provided biomass concentration and polymer content of up to 23 g/L and 56%, respectively, was observed in a culture of the natural strain *Cupriavidus necator* [50]. In the culture of the natural strain *Cupriavidus necator* ATCC 25207, the applicability of untreated sugar beet molasses and molasses hydrolyzed with acid or invertase for PHB production in various cultivation modes was evaluated [58]. During cultivation in flasks on untreated molasses, the biomass concentration was 2.1–3.9 g/L and the polymer content was 4.4–4.9%. The values for hydrolyzed molasses were higher, 5.5 g/L and 43.2%, respectively. When cultivating bacteria in a fermenter, the concentration of biomass and polymer was 29 g/L and 53%.

In general, given the promise of molasses as a potential source of carbon in PHA production and the ambiguity and wide scatter of available data, it is necessary to study the processes of PHA biosynthesis in conjunction with methods for pretreatment of molasses. The purpose of this work is to study PHA production by a natural strain of *Cupriavidus necator* using sugar beet molasses as the main growth substrate

## 2. Materials and Methods

### 2.1. Microorganisms

The polymers were synthesized using the *Cupriavidus necator* B-10646 wild strain, which is registered in the Russian National Collection of Industrial Microorganisms (NCIM) [62]. The strain is capable of synthesizing valuable PHA copolymers at high contents and is tolerant to precursor substrates. The strain has a broad organotrophic potential and can utilize such carbon sources as sugars, amino acids, organic acids, alcohols, CO_2_, and CO. The strain synthesizes PHA copolymers composed of short- and medium-chain-length monomers [63].

### 2.2. Nutrient Medium

The Schlegel medium with CO(NH_2_)_2_ as a source of nitrogen with 1.0 g/L [26] was taken as the basis. The medium had the following composition: Na_2_HPO_4_·12H_2_O–9.1; KH_2_PO_4_–1.5; MgSO_4_·7H_2_O–0.2; C_6_H_5_O_7_Fe·nH_2_O–0.025 (g/L), and the trace element solution (3 mL of standard solution per 1 L of the medium). The solution contained H_3_BO_3_–0.288; CoCl_2_·6H_2_O–0.030; CuSO_4_·5H_2_O–0.08; MnCl_2_·4H_2_O–0.008; ZnSO_4_·7H_2_O–0.176; and NaMoO_4_·2H_2_O–0.050; NiCl_2_–0.008 (g/L). A nitrogen concentration of 0.4–0.6 g/L, limiting the growth of bacteria, and was adopted at the first stage of the two-stage mode of PHA synthesis. The main carbon source was glucose (China, purity 98%). A complex sugar-containing substrate was represented by sugar beet molasses and its derivatives. The initial molasses (manufactured by Ertilsky Sakhar Ltd., Ertil, Russia) contained 79.6 and 46.8 solids, 1.5–2.0 total nitrogen, 4.0–7.0 betaine, and 6.0–11.0 conductometric ash (% by weight). To synthesize PHA copolymers, the cell culture was supplemented with precursors of different monomers: potassium propionate and valerate, hexanoate, or ε-caprolactone (Sigma-Aldrich, Saint Louis, MO, USA) at concentrations of 1–2 g/L.

### 2.3. Hydrolysis of Sucrose to Molasses

Molasses was subjected to hydrolysis to transform sucrose into hexoses that are accessible to bacteria. For this, acid and enzymatic hydrolysis was carried out. The molasses was preliminarily twice diluted with water, to a solid concentration of 40%. The degree of hydrolysis (D) was determined by the formula:

D (%) = S_v_/C_0_ × 100%,

where S_v_—the total amount of reducing sugar (glucose + fructose) in g/L, C_0_—initial concentration of sucrose.

The acid hydrolysis of sucrose in molasses was carried out with a solution of 1.5 N hydrochloric acid (HCl): 5 mL of HCl was added to 100 mL of a 500 g/L sucrose solution and kept at 90 °C in a water bath for 60 min. Then, the solution was cooled to room temperature for 30 min and the pH was adjusted to 7 with 1M KOH solution [57]. The enzymatic hydrolysis of sucrose in molasses was carried out with the extracellular enzyme *β*-fructofuranosidase, which obtained by the autolysis of *Saccharomyces cerevisiae* yeast cells [64]. To do this, 31.5 g of pressed baker’s yeast was mixed in 50 mL of water (or 6.3 g of dry, previously aged yeast in 100 mL of warm water for 15 min) and the baker’s yeast was kept at 50 °C for 20 h on a magnetic stirrer. The resulting autolysate was centrifuged at 6000 rpm, and the precipitate was suspended in 100 mL of an aqueous solution of 0.15 KCl and centrifuged again at 6000 rpm. The suspension and centrifugation of the precipitate were repeated two more times until a clear, colourless centrifuge was obtained. The precipitate obtained, consisting mainly of yeast cell walls, was suspended in 200 mL of distilled water and centrifuged at 6000× *g* for 10 min. Next, the precipitate was added to 250 mL of molasses solution, which was previously diluted 1:1 with distilled water, and the pH was adjusted to 4.5 by adding concentrated sulfuric acid and was kept at 55 °C for 24 h on a magnetic stirrer. The resulting hydrolyzed molasses was neutralized with a KOH solution. The clarification and removal of components other than sugars in molasses were carried out after sucrose hydrolysis according to the method [65]. Molasses with hydrolyzed sucrose were heated to 55 °C on a stove with a stirrer and 33% hydrogen peroxide was added in an amount of 7% by volume and was kept for 24 h. Then, the molasses was cooled and centrifuged at 6000 rpm for 5 min. The supernatant was decanted, the weight of the dry residue was determined, and the optical density was measured at 600 nm.

### 2.4. Study of the Composition of Molasses

The concentration of fructose was determined using the resorcinol method [66]. The concentration of glucose was determined spectrophotometrically at 490 nm by the glucose oxidase method using a Fotoglucoza kit (Impact Ltd., Moscow, Russia). The nitrogen concentration in the culture medium was analyzed at different time points using a photometric method with Nessler’s reagent. To measure the concentrations of major elements (S, K, Mg, P, Na, Ca) and trace elements, samples of the culture medium were taken periodically and measured using inductively coupled plasma atomic emission spectroscopy in an ICAP–6000 Thermosystem (Thermo Electron Corporation, Waltham, MA, USA).

### 2.5. Technique and Methods of Cultivation of Microorganisms

For the cultivation of bacteria, various types of laboratory fermentation equipment were used. Cultivation was carried out in 1 L and 2 L glass flasks, with a filling of 50% on a thermostatically controlled Incubator Shaker Innova^®^ series 44 (New Brunswick Scientific, Edison, NJ, USA). Process scaling is implemented in fermenters. We used an 8 L BioFlo 110 (New Brunswick Scientific, Edison, NJ, USA). We cultivated bacteria in a batch mode, observing the conditions developed earlier for PHA biosynthesis. The inoculum was obtained from the museum culture under strictly sterile conditions in glass flasks and used for the inoculation of fermentation apparatuses. The starting concentration of cells in the medium when using flasks was not less than 0.1–0.2 g/L, and it was not less than 1.0–2.0 g/L when using fermenters. Bacteria were cultivated on a saline Schlegel medium at an initial concentration of C-substrate of 10–15 g/L and a medium temperature of 30 °C. A periodic two-stage process was used with cells grown under limited nitrogen supply in the first stage and no nitrogen in the second. In high-density fermentation cultures, as the cell concentration increased, the medium was periodically replenished with concentrated solutions of C-substrate and mineral elements using peristaltic dosing pumps.

### 2.6. Methods for Controlling the Parameters of the Bacteria Cultivation Process in the PHA Synthesis Mode

The culture samples were periodically taken for analysis during cultivation. The dynamics of cell growth in culture was detected by the optical density of the bacterial suspension at a wavelength of λ = 440 nm (UNICO 2100 photoelectric calorimeter, Dayton, NJ, USA). The bacterial biomass concentration (X, g/L) was estimated by weight drying at 105 °C for 24 h for washed cells and preliminarily centrifuged at 6000 rpm.

The intracellular PHA content in bacterial cells and its composition were determined by chromatography of fatty acid methyl esters after methanolysis of biomass samples on an Agilent Technologies 7890A chromato-mass spectrometer with a 5975C mass detector (Agilent Technologies, Santa Clara, CA, USA). Methanolysis of the samples was conducted as follows: 1 mL chloroform, 0.85 mL methanol, and 0.15 mL concentrated sulfuric acid were added to a 4.0–4.5 mg polymer sample and boiled under reflux condensers for 160 min. At the end of the methanolysis reaction, 1 mL distilled water was added to the flask. After phase separation, the lower organic phase containing hydroxyalkanoic acid methyl esters was analyzed. Benzoic acid was used as an internal standard to determine total intracellular PHA [67].

The concentration of cell biomass in culture (X, g/L), the concentration and content of PHA (g/L and in % of the weight of dry matter content), the specific rates of bacterial growth and PHA synthesis (µ, h^−1^), the productivity of the biosynthesis process in terms of biomass and polymers (P, g/L × h), and the economic coefficient in terms of biomass and polymers (Yg/g) were recorded using conventional methods. Microorganism cultivation techniques and microbial culture control methods had been previously described in detail [68].

### 2.7. Physicochemical Properties of PHAs

PHA was extracted from the cell biomass, which was previously separated from the culture fluid by centrifugation in an AvantyJ-HC centrifuge (BeckmanCoulter, Indianapolis, IN, USA). Then, the biomass was dried in an LP10R freeze dryer (ilShinBioBase, Dongducheon-si, Korea) to a residual moisture content of 5%. The extraction was carried out in two stages: at the first stage, the biomass was degreased with ethyl alcohol; at the second stage the polymer was extracted with methylene chloride. Thereafter, the polymer solution was filtered, and the polymer was precipitated with ethanol.

The physicochemical properties of PHAs were examined by using high performance liquid chromatography, X-Ray structure analysis, and differential scanning calorimetry. The methods and instruments had been described in detail before. The molecular-weight properties of PHAs were examined with gel permeation chromatography and a DB-35MS column (Agilent Technologies 1260 Infinity, Santa Clara, CA). The weight average molecular weight (M_w_), number average molecular weight (M_n_), and polydispersity (Ð) were measured using polystyrene standards (PS) (Agilent Technologies, Santa Clara, CA, USA). The melting point (T_melt_) and thermal degradation temperature (T_degr_) were measured using a DSC-1 differential scanning calorimeter (Mettler Toledo, Schwerzenbac, Switzerland) and TGA (Mettler Toledo, Schwerzenbac, Switzerland), respectively. The melting point and thermal degradation temperature were determined from endothermic peaks in thermograms. The thermograms were analyzed using the STARe v11.0. software (Mettler Toledo, Schwerzenbac, Switzerland). The X-Ray structure analysis was performed to determine the crystallinity of copolymers employing a D8 ADVANCE X-Ray powder diffractometer equipped with a VANTEC fast linear detector (Bruker AXS, Karlsruhe, Germany). The degree of crystallinity (C_x_) was calculated as a ratio of the total area of crystalline peaks to the total area of the radiogram (the crystalline + amorphous components). The calculations were done by using the Eva program of the diffractometer software.

### 2.8. Statistics

The statistic processing of the experimental data was carried out by conventional methods using the standard Microsoft Excel software package. The average values of the results were determined. The deviations from the mean for each result variance, the standard deviation of an individual result, and the standard deviation of a mean result were calculated.

## 3. Results and Discussion

In the studied sample of molasses, the content of dry substances was 79.6% and the content of sugars was 52.3%. Based on the initial composition of molasses, which contains sucrose and is not available for growing strains of *Cupriavidus necator*, an attempt was made to adapt the strain to this substrate. The original molasses was diluted with tap water to a physiologically acceptable concentration of sucrose for bacteria (10–20 g/L). In the original molasses, the content of glucose and fructose (C-substrates utilized by this strain for the growth and synthesis of PHA) is extremely low (1.6 and 1.7 g/L, respectively). After sterilization in an autoclave, as a result of the partial hydrolysis of sucrose, the content of glucose and fructose in molasses increased to 7.5 and 7.6 g/L, respectively. Based on the previously determined limits of physiological action for this strain of fructose (not higher than 15 g/L) and glucose (up to 30 g/L), as well as the known data on high minerals and other components of molasses that inhibit the growth of many microorganisms [58], molasses was diluted 20 times with water (50 mL per 1 L of medium). However, an attempt to adapt the strain to native molasses as the only C-substrate did not give positive results, despite the fact that dozens of bacteria passages were carried out. An extremely low concentration of bacterial biomass, below 0.5–0.7 g/L, was obtained due to the utilization of hexoses, which are included in the molasses. This is consistent with published results [58,60] that showed an extremely low biomass concentration and polymer production in *Ralstonia eutropha* PTCC1615 and *Cupriavidus necator* ATCC 25207 strains using raw sugar cane molasses or beets.

Therefore, we decided to subject the molasses to preliminary hydrolysis to transform sucrose into glucose and fructose, which are utilized by the studied bacterial strain *C. necator* B-10646.

### 3.1. Characteristics of the Hydrolysates of Sugar Beet Molasses

The characteristics of the hydrolysates of sugar beet molasses are given in Table 1. The most complete conversion of sucrose to hexoses (88.9%), and with the almost equal formation of glucose and fructose, was obtained with enzymatic hydrolysis at a significantly longer time (24 h) compared to chemical hydrolysis (3 h) (Table 1).

During acid hydrolysis, only half of the sucrose was hydrolyzed into hexoses, with an uneven 3:1 ratio of glucose and fructose. The acid hydrolysis of sucrose is known to be accompanied by the formation of undesirable side products, such as formic and acetic acids, hydroxymethylfurfural (HMF), monosaccharide anhydrides, and humic substances, which can inhibit the growth of microorganisms [57,69]. Enzymatic hydrolysis avoids the oxidation of fructose, and the resulting molasses contains almost equal amounts of glucose and fructose. Based on the results obtained, an enzymatic method for the hydrolysis of molasses was used.

Further, the resulting molasses hydrolysate was subjected to clarification using H_2_O_2_ to precipitate an excess amount of mineral substances [65]. The results of the analysis of the chemical composition of the initial molasses and the resulting hydrolysate are presented in Table 2. The concentration was selected (7 mL H_2_O_2_/100 mL hydrolysate), at which point a part of the element’s precipitates, which are not biogenic for bacteria—including 95% Ca, 78% Cr, 75% Sr—were used. There was also a slight decrease in the concentration of a number of trace elements: B, Co, Mn, Mo, and Ni (Table 2). The results of the mineral composition analysis of the initial molasses and molasses after clarification are presented in Table 2. A similar treatment of molasses was carried out using activated carbon [56]. This treatment made it possible to reduce the content of calcium, iron, silicon, phosphorus, and titanium without changing the sugar content.

We performed an analysis of the balance in chemical composition of the obtained molasses hydrolysate with the physiological needs of bacteria based on the data obtained earlier in the study of the mineral nutrition of hydrogen-oxidizing bacteria [70]. The specific needs of the culture of hydrogen-oxidizing bacterium *A. eutrophus* Z1 in nitrogen and mineral elements are N = 125 ± 5; P = 18 ± 0.5; S = 6.5 ± 0.5; and K and Mg at 5.0 ± 0.5 mg/g, which was calculated for the synthesis of 1.0 g of CDW. It is important to note that the physiological requirements of mineral elements for bacteria are very wide, and inhibition of cell growth by an excess of macro- and microelements occurs when their concentration in the culture medium increases above 500–700 mg/L. Therefore, the excess content of some macro-elements in molasses did not cause concern.

Based on the results of comparing the content of C-substrate (glucose + fructose) and mineral macro- and microelements in the standard Schlegel medium—and the obtained sugar beet molasses hydrolysate—we concluded that the sugar content is excessive (by more than an order of magnitude), and the composition of the molasses hydrolysate is balanced for most mineral elements to provide the physiological needs and unlimited growth of bacteria. The exceptions were nitrogen and phosphorus. The amount of nitrogen is excessive, which stimulates cell growth and the synthesis of the main intracellular components (protein and nucleic acids). Obtaining the highest content of PHA (up to 80–85% and higher) in the culture of *C. necator* B-10646 and the closely related strains is possible only with unbalanced growth and by limiting the growth of bacteria for this element [12]. At the same time, the phosphorus content in the molasses hydrolysate is low (about 100 mg/L), which can provide no more than 6.0 g of cell biomass. Given the need to dilute the hydrolyzed molasses to reduce the excess concentration of sugars and nitrogen, a deficiency of this element will limit cell growth. Therefore, a source of phosphorus must be added to the molasses hydrolysate when it is used as the basis of a nutrient medium for bacteria. The total content of elements in molasses after the hydrolysis procedure did not show in what forms they are present and how accessible they are for assimilation by bacteria.

### 3.2. Production Characteristics of Cupriavidus necator B-10646 Culture When Grown on Molasses Hydrolysate

**Figure 1:**
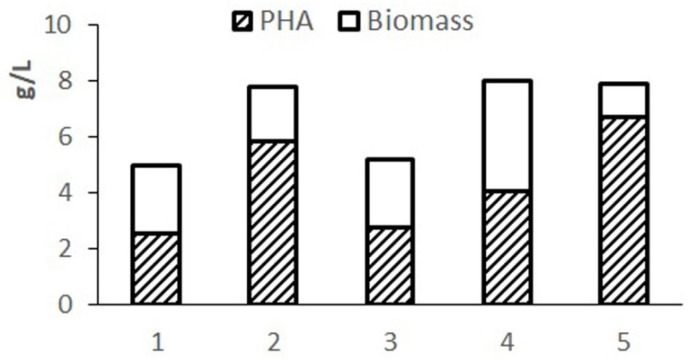
Indicators of the culture of *Cupriavidus necator* B-10646 when grown on molasses hydrolysate and Schlegel’s medium: 1-, 2-, 3-, 10-, 20-, 30-fold dilution of molasses hydrolysate, respectively; 4-complete Schlegel medium; 5-with 50% nitrogen content. Biomass concentration (X, g/L), polymer (% of CDW).

Preliminary testing of the balance and availability of hydrolyzed molasses components for the studied bacterial strain was carried out by varying the composition of the nutrient medium. The results of cultivating bacteria in 1.0 L flasks using various options for diluting molasses hydrolysate in comparison with the Schlegel medium are given in Figure 1. Phosphorus was introduced into hydrolyzed molasses in the form of magnesium phosphate.

The complete Schlegel medium provides a concentration of cell biomass up to 7–8 g/L but limits the accumulation of the polymer due to excess nitrogen in the medium. The highest biomass concentration and polymer content were obtained on Schlegel’s medium with a halved nitrogen content. A close result is provided by using a 20-fold dilution of molasses hydrolysate. When using a less diluted hydrolysate, cell growth and polymer synthesis are inhibited due to excess sugars. A highly diluted hydrolysate (30-fold dilution) limits cell growth with less of an effect on polymer production due to reduced sugar content. The results showed the need to adjust the composition of the nutrient medium based on molasses hydrolysates in terms of sugars and nitrogen.

**Figure 2:**
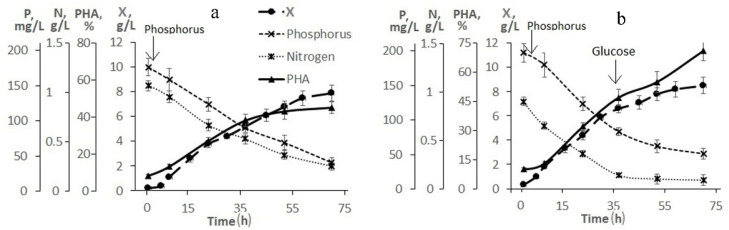
Production parameters of bacterium *Cupriavidus necator* B-10646 when grown on hydrolyzed sugar beet molasses in 2 L glass flasks under different scenarios of its use: (**a**)—20-fold dilution of molasses hydrolysate; (**b**)—30-fold dilution of molasses hydrolysate with the addition of phosphorus and glucose to the culture (indicated by arrows).

Figure 2 illustrates the need and result of adjusting the composition of the medium based on molasses hydrolysate. As follows from Figure 2a, when using a 20-fold dilution of molasses hydrolysate, the initial concentrations of sugars (fructose + glucose) and nitrogen were 26.0 and 1.2 g/L, respectively. The concentration of phosphorus was 5.4 mg/L, therefore, it was introduced into the culture in the first hours of the bacterial cultivation process. The nitrogen concentration in the culture medium decreased. However, even during the entire second stage, nitrogen was determined in culture, which indicated an excess of this element and a negative effect on the accumulation of the polymer in cells. The intracellular content of the polymer increased by the end of the first stage to 45% and remained practically unchanged (at the level of 50%) until the end of the cultivation process (68–70 h). The bacterial biomass concentration was about 8.0 g/L.

To increase the production indicators of the culture and eliminate the excess nitrogen content in the bacterial culture, a process for growing bacteria with an adjustment to the composition of the medium during culture development is proposed (Figure 2b). In this variant, a 30-fold dilution of molasses hydrolysate was used. Therefore, the initial concentrations of sugars and nitrogen in it were reduced to 18.0 and 0.8 g/L, respectively. The phosphorus concentration did not exceed 3.5 mg/L (it was added to the culture in the first hour of the process). The nitrogen concentration dropped during the first stage, but it was preserved in the culture at the second stage. At the beginning of the second stage (34–35 h), the culture was supplemented with glucose. Glucose was added because the initial sugar content was reduced, and molasses additives were not used to eliminate excess nitrogen. The bacterial biomass concentration was 8.0 g/L by the end of the experiment and the intracellular polymer content was 75%. It should be noted that hexoses (fructose and glucose) were utilized from the medium by the cells simultaneously; no diauxia was observed. The proposed mode provided a productive process under the conditions of a low-diluted flask culture with indicators in terms of X and g/L comparable to the studied strain on pure sugars, however, with a slight decrease in the polymer content (up to 70–75%).

A comparison of the obtained results with published ones showed the following. Natural strains, representatives of the taxon *Cupriavidus* (formerly *Ralstonia*), when grown on hydrolyzed sugar cane molasses obtained by various methods and can provide different indicators of PHA production when growing bacteria in flasks. For example, the indicators were low, and the polymer content was at the level of 27% at a bacterial biomass concentration of 2.9 g/L [57]. Similarly, in [60], *Ralstonia eutropha* PTCC1615 bacterium, when grown in flasks, accumulated no more than 30% polymer on the cane and sugar beet hydrolysates at a biomass concentration of about 3.0 g/L. When grown on treated molasses, the natural strain *Cupriavidus necator* ATCC 25207 synthesized up to 43.2% polymer at a low biomass concentration of 5.5 g/L [58]. A higher biomass concentration of *C. necator* DSM 545 (about 10 g/L) was obtained in [61], while the polymer content did not exceed 13%.

A similar spread of indicators is typical for genetically engineered strains, which can accumulate PHA on raw molasses with different biomass concentrations—from a relatively low polymer content, about 24–32%, at X values below 5.0 g/L [55] and 46.9% and X = 4.4 g/L [56] for higher values. For example, up to 40% PHA was synthesized in 36 h at a bacterial biomass concentration of 8.0 g/L [54] and 65% of the polymer was synthesized in 72 h at X = 7.0 g/L [55]. 

The subsequent modification of the proposed mode of cultivating bacteria (Figure 2b) and scaling the process using fermenters ensured the implementation of a productive process in terms of the biomass concentration and PHA content. However, due to obtaining a significantly higher biomass concentration when growing bacteria in a fermenter compared to flask cultures, the medium composition must be adjusted for the mode of controlled feeding of the culture with all nutrients. At the same time, current concentrations in the culture of sugars, nitrogen, and phosphorus, which is sharply deficient, should be under special control. Preliminary experiments in the 8 L BioFlo fermentation complex with a fill factor of 0.5 and a mass transfer coefficient of KLa 200–800 h^−1^ showed the possibility of obtaining a biomass concentration on diluted molasses hydrolysates of up to 10–15 g/L.

**Figure 3:**
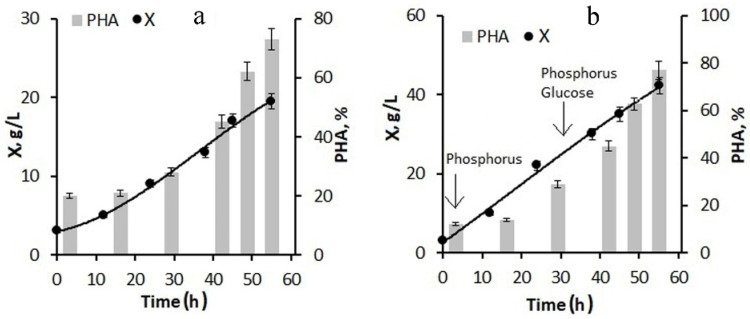
Production parameters of the bacterial culture *Cupriavidus necator* B-10646 when grown on hydrolyzed sugar beet molasses in the 8 L fermenter under different versions of feeding the culture with nutritive elements: (**a**)—20-fold dilution of molasses hydrolysate; culture feeding with phosphorus at the first stage, and feeding with phosphorus + glucose at the second stage; (**b**)—20-fold dilution of molasses hydrolysate with feeding the culture with phosphorus and glucose at the first and second stages (indicated by arrows).

When using 20-fold molasses hydrolysate and adding only phosphorus to the culture at the first stage of the process, the biomass concentration by the end of the first stage was about 10–15 g/L with a polymer content of about 40–45%. The maximum specific growth rate was reached at 20 h of cultivation and was 0.05 h^−1^; the specific rate of polymer synthesis reached its maximum at the end of the first stage and the beginning of the second stage of cultivation and was 0.07 h^−1^. The culture was also supplied with glucose, the consumption of which sharply decreased as the growth and division of cells stopped and the polymer accumulated in them. With this background, the specific growth rate of bacteria decreased to 0.01 h^−1^. Glucose supplementation was used instead of molasses to avoid excess nitrogen in the culture, which limited polymer accumulation (Figure 3a). By the end of the second stage, the bacterial biomass concentration was 20 g/L; the polymer content was about 75%.

The next version of the process for increasing the concentration of the bacterial biomass included feeding the bacterial culture with glucose in addition to phosphorus at the first stage (Figure 3b). The phosphorus supply to the culture began from the first hours of the bacterial cultivation process; the glucose supply began in the middle of the first stage. As a result, by the end of the first stage, the bacterial biomass concentration was 25–30 g/L, the specific growth rate of bacteria was 0.08 h^−1^, and the specific rate of polymer synthesis was 0.13 h^−1^. At the end of the experiment, the biomass concentration reached 42 g/L at a polymer content of about 77–80%. Thus, the combination of molasses hydrolysate with glucose feeding of the culture can significantly increase the biomass concentration while maintaining a high polymer content.

**Figure 4:**
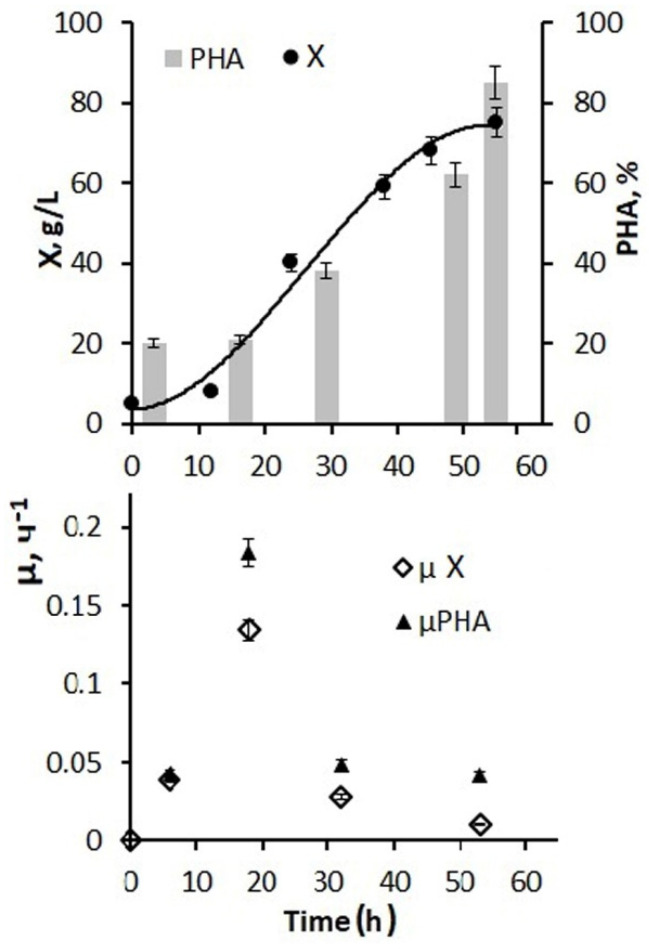
Production parameters of *Cupriavidus necator* B-10646 culture when cultivated in the 8 L fermenter using 20-fold dilution of molasses hydrolysate, concentrated inoculum (5 g/L), and a larger amount of glucose and phosphorus supplementation as a source of carbon, nitrogen, and mineral elements at the first stage with feeding with phosphorus and glucose; at the second stage—in a nitrogen-free medium with culture fed with glucose and phosphorus (feeds are shown by arrows): the concentration of the total cell biomass in the culture (X, g/L) and the polymer in the cells (PHA% of CDW); specific cell growth rate (µX h^−1^) and specific polymer synthesis rate (µPHA, h^−1^).

Thus, using diluted hydrolyzed molasses, due to excess nitrogen, does not allow for high concentrations of biomass to be obtained due to the low sugar content. The addition of glucose at the first stage of the process has a positive effect. Figure 4 shows the results of using a larger amount of glucose and phosphorus additions and a 5 g/L inoculum concentration for the fermenter inoculation. By the end of the first stage, it was possible to increase the bacterial biomass concentration to 45–50 g/L, and the total biomass concentration for the entire process increased to 75–80 g/L. At the same time, the specific rate of bacterial synthesis increased to 0.12 h from the beginning of the process and decreased to 0.01 h^−1^ by the end of cultivation.

Active cell and biomass growth in the exponential phase were accompanied by active consumption of both hexoses, and the average value for this stage of the process was 2.7–2.9 g/g·h. Further, at the second stage, in the presence of a decrease in growth characteristics, the consumption of supplied glucose decreased and by the end of the process amounted to 0.05–0.08 g/g h. The productivity of the process for biomass was 1.48 g/L·h. For the polymer, it was 1.23 g/L·h. The values of the effectiveness for using the C-substrate, which were estimated by the economic coefficient, are somewhat inferior to those obtained in this culture on individual sugars. This is a consequence of the presence of inhibitory components in the composition of molasses, which did not affect the concentration of the bacterial biomass under nitrogen-limited cell growth, but compensatory increased substrate consumption was performed to reduce the inhibitory effect. Therefore, the economic coefficient for C-substrate (Y g/g) for biomass was 0.29 and for PHA it was 0.23.

Thus, the developed mode of cultivating bacteria in a fermenter using molasses hydrolysates in combination with glucose and phosphorus additives ensured the implementation of a productive process of PHA synthesis. Depending on the number of glucose additions to molasses hydrolysates and the sugar ratio of these two sources (from 1:1 to 1:10), it is possible to obtain a bacterial biomass concentration from 20 to 80–85 g/L with a polymer content of about 80%. 

Comparing the results obtained with publications showed that they are superior to the data of some works but also inferior to individual data. The spread of data on the production parameters of the PHA synthesis process is mainly related to the type of producer strain used. In cultures of natural strains of the taxon *Cupriavidus* (*Ralstonia*), as a rule, the results are inferior to those obtained in this work. Thus, when growing the natural strain *Ralstonia eutropha* ATCC 17699 and using untreated molasses and vinasse as a carbon substrate for 50 h of cultivation in a 5 L fermenter at a ratio of molasses and vinasse such as 25:75, the biomass and polymer concentrations were low and amounted to 3.9 and 2.7 g/L, respectively. In the original molasses, sucrose was the main sugar (60–63%); in vinasse, the content of sucrose, glucose, and fructose was 3.7, 2.4, and 5.5 g/L, respectively [71]. When cultivating the natural strain *Cupriavidus necator* ATCC 25207 in a 5 L fermenter for 52 h, the maximum biomass concentration (29 g/L) and polymer content (53%) were obtained on a medium containing molasses hydrolyzed with sulfuric acid. The initial concentration of the hydrolyzed molasses solution was 55 g/L and the total concentration of the added molasses solution was 112 g/L [58]. In the culture of the natural strain *Cupriavidus necator* grown on molasses hydrolysate in a 7.5 L fermenter for 50 h, the biomass concentration was 23 g/L with a low polymer content of 56% [50]. Higher rates are typical for processes using recombinant strains capable of metabolizing molasses sucrose. In the culture of the recombinant strains of *Ralstonia eutropha* containing the sacC gene from *Mannheimiasuc ciniciproducens*, encoding β-fructofuranosidase, which can hydrolyze sucrose into glucose and fructose in a nutrient medium in a 2.5 L fermenter, led to the following indicators being achieved in 36 h: biomass of about 30 g/L, polymer content 82.5% [56]. A recombinant strain of *Klebsiella aerogenes* carrying genes for PHB synthesis from *Alcaligenes eutrophus* was grown on molasses as the only carbon source in a 10 L fermenter for 32 h. The concentration of the bacterial biomass was 37 g/L and the polymer content was 70% [53]. The highest result was obtained by Arikawa et al. 2017 [54]. A recombinant strain of *C. necator* containing genes for utilizing sucrose from *E. coli* in a 5 L fermenter provided a bacterial biomass concentration of 113 g/L in 65 h with a polymer content of 70–80%.

The obtained results and published data allow us to conclude that molasses is a promising substrate for PHA production, including highly productive representatives of the *Cupriavidus* taxon.

### 3.3. Properties of PHA Synthesized from Sugar Beet Molasses

Properties of PHAs are determined, primarily, by the structure of side chains in the polymer carbon chain and the distance between the ester groups in a molecule. The valuable properties of PHA producers include the ability to synthesize more technologically advanced copolymers ((P(3HB-*co*-3HV), P(3HB-*co*-3HHx), and P(3HB-*co*-4HB), etc.)) in addition to the highly crystalline homopolymer of 3-hydroxybutyric acid [P(3HB)]. The synthesis of PHA copolymers is usually achieved by supplementing the culture medium with different additional precursor carbon sources (valerate, hexanoate, ε-caprolactone, etc.), which are toxic to bacteria and, thus, impair the total productivity of the biosynthesis and reduce polymer yields. Therefore, it is important to find and/or engineer strains that would be tolerant to these compounds. Depending on the structure of the C-chain, the known types of PHA are divided into three groups: short-chain, formed by monomers with a C-chain length from C3 to C5, medium-chain (C6–C16), and long-chain (C17 and above) [72]. A carbon source is one of the cultivation conditions considerably influencing the chemical composition and properties of PHAs. As a rule, the synthesis of copolymeric PHAs is realized using genetically engineered strains. The ability of individual natural strains to synthesize copolymeric PHA, including those containing short- and medium-chain monomers, was first shown in cultures of natural strains *R. eutropha* B5786 and *R. eutropha* H-16 [32,73,74]. 

The authors of these works added valerate, hexanoate, and octanoate as precursors for the monomers other than 3-hydroxybutyrate to bacterial cultures in the PHA synthesis mode and obtained some copolymers.

In the composition of hydrolysates of sugar beet molasses obtained by the enzymatic method, the presence of propionic (up to 0.3%) and n-valeric (up to 0.2%) acids, which are precursors for the synthesis of the monomer 3-hydroxyvalerate (3HV), were found in small concentrations. It prompted us to analyze the chemical composition of the polymer synthesized by *C. necator* B-10646 on this substrate. It was shown that regardless of the cultivation technique and intensity of mass transfer (glass flasks installed in a shaker-incubator or fermentation complexes), the synthesized polymer is a P(3HB-*co*-3HV) copolymer with minor (0.3–0.5 mol.%) inclusions of 3HV monomers (Table 3).

The literature analysis has shown that the vast majority of data described the synthesis of the P(3HB) homopolymer on the growth of natural and recombinant strains on molasses or its hydrolysates. The synthesis of P(3HB-*co*-3HV) copolymers was recorded in [75] when the bacteria *Bacillus cereus* RCL 02 (MCC 3436) was grown on sugar cane molasses; the copolymer content was high (up to 83.5%) and the content of 3HB monomers varied from 1.2 to 12.4 mol.% depending on the concentration of molasses in the medium. There are also examples of the synthesis of P(3HB-*co*-3HHx) copolymers on molasses in cultures of genetically engineered producers, *C. necator*, which contain genes for the metabolism of sucrose from *E. coli* [54], and *C. necator* 2058/pCB113 [55]. The ability to synthesize a poly(3-hydroxybutyrate-*co*-lactate) [P(3HB-*co*-LA)] copolymer was found by a recombinant *Ralstonia eutropha* strain containing the sacC gene from *Mannheimia succiniciproducens* encoding *β*-fructofuranosidase [56].

It was shown that the addition of precursor substrates (potassium propionate or valerate, ε-caprolactone, or hexanoate at a concentration of 1.0–2.0 g/L) to the culture of *C. necator* B-10646 grown on the hydrolysates of sugar beet molasses was accompanied by the synthesis of copolymers of various compositions P(3HB-*co*-4HB), P(3HB-*co*-3HV) and P(3HB-*co*-3HHx) with more significant inclusions of second monomers other than 3HB. The additions were made during the period of active polymer synthesis. This is 10–12 h from the beginning of the cultivation process. The highest level of incorporation of 4HB, 3HV, or 3HHx monomers into the 3-hydroxybutyrate chain occurred 10–15 h after adding the precursor at the end of the first stage of bacterial cultivation. Further, the content of monomers other than 3HB decreased, which is due to the activity of enzymes of intracellular degradation of the polymer, primarily PHA-depolymerase. 

Properties of PHA with different composition are presented in Table 3. 

The properties of P(3HB-*co*-3HV) with a minor content of 3HV monomers grown on molasses hydrolysate (without the addition of precursors) corresponded to the values characteristic of the P(3HB) homopolymer. It includes a high degree of crystallinity (76–80%), the melting temperature, and thermal degradation values (173–180 and 276–287 °C, respectively), with a gap between the values of T_melt_ and T_degr_ of at least 100 °C. The samples are also characterized by a variability in the molecular weight characteristics and relatively low polydispersity values (less than 3.0).

All PHA samples synthesized on molasses hydrolysate with the addition of precursors and having significant inclusions of second monomers (from 5–6 to 10–16 mol.%) are characterized by a slight decrease in molecular weight and an increase in polydispersity. The most important parameter of polymer materials is the degree of crystallinity: the ratio of the crystalline to amorphous phase. The percentage of the monomers other than 3HB affected the degree of crystallinity of the polymers, which decreased as the fraction of the second monomer increased. That was more noticeable in the P(3HB-*co*-4HB) specimens, whose Cx decreased to 45 and 38% at 4HB 6.5 mol.% and 12.0 mol.%, respectively. The P(3HB-*co*-3HV) and P(3HB-*co*-3HHx) copolymers exhibited a similar, although less obvious, trend. The degree of crystallinity of P(3HB-*co*-3HV) containing 3HV 8.0 and 16.5 mol.% was 67 and 55%, respectively; the degree of crystallinity of P(3HB-*co*-3HHx) containing 3HHx 5.6 and 10.3 mol.% was 70 and 68%, respectively. Thus, in all PHA copolymers, the crystalline phase decreased while the amorphous, disordered regions increased, indicating better processability of these polymers. 

Double peaks are present on the thermograms of the copolymers (Figure S1), which indicate the presence of crystals of various shapes or sizes in the polymer mixture. This confirms the presence of a mixture of homopolymers and copolymers. In general, the properties of the studied samples correspond to the large array of published data characterizing the relationship between the set and ratio of monomers in copolymer PHA and their basic physicochemical properties and are consistent with the results of many other studies [9,10,11,14,15].

Other authors made a similar conclusion while studying the PHA properties, which were synthesized by various strains on molasses and its derivatives. Thus, analyzing the properties of the P(3HB) homopolymer synthesized when grown on molasses of various origins or its derivatives, the authors [55,58] concluded that the properties of the samples were similar to commercial analogues of poly(3-hydrohybutyrate). Thus, M_w_ was determined, levelling from 384 to 670 kDa and polydispersity was determined, levelling from 1.8 to 3.81. One of the polymer samples had two melting peaks: 142.08 and 159.43 °C. In others, for T_melt_ it was 172.43 °C and the beginning of T_degr_ was 262.0 °C. At the same time, P(3HB) samples synthesized on molasses by strains of other taxa showed some differences in the properties of P(3HB). Thus, P(3HB) synthesized by *Pseudomonas fluorescens* on molasses had low values of the degree of crystallinity (9.5%), which was determined based on enthalpy measurements; at the same time, in a commercial sample, the Cx value was also significantly lower than the generally accepted values [76]. The P(3HB) sample synthesized by the *Bacillus megaterium* JHA strain on molasses had two melting peaks: 136.41 °C and 158.04 °C. While measuring the molecular weight characteristics of the polymer, the presence of two peaks in the chromatogram was shown. The first one was in the region of M_n_ = 47.84 kDa and M_w_ = 71.65 kDa with a polydispersity of 1.49; the second peak was M_n_ = 0.08 kDa and M_w_ = 0.55 kDa with a polydispersity of 6.85. The presence of both peaks confirms that the resulting polymer P(3HB) is a mixture [77].

There is little information about the synthesis of molasses and the study of copolymer PHA. Thus, the P(3HB-*co*-3HV) copolymer synthesized by *B. cereus* RCL 02 on sugar cane molasses was studied and the content of 3HV monomers varied from 1.2 to 12.4 mol.% depending on the concentration of molasses [75]. The maximum inclusion (12.4 mol.%) was obtained using 4% molasses. T_degr_ of the copolymer (in terms of 50% weight loss) was fixed at 263.14 °C. The melting point of the polymer was 169.72 °C. The P(3HB-*co*-3HHx) copolymer characterized by Purama et al. 2018 had M_w_ values in the range of 580–830 kDa; the highest M_w_ was determined for the sample containing 5 mol.% of 3HHx [55]. Two peaks of T_melt_ (142.08 and 159.43 °C) and slightly lower temperature characteristics were found. 

The published data and the results of the presented work allow us to summarize the important possibility of synthesizing more technologically advanced PHA copolymers using molasses and precursors of 3HV, 3HHx, and 4HB monomers as C-substrates.

## 4. Conclusions

We studied beet molasses as the main growth substrate for the PHA synthesis. Acid and enzymatic hydrolysis were carried out to transform molasses sucrose into hexoses that were accessible to the studied strain-producer. Enzymatic hydrolysis using β-fructofuranosidase provided the complete conversion of sucrose (88.9%) to hexoses with an almost equal ratio of glucose and fructose, in the level of about 35–45% of the obtained monosaccharides. The chemical composition analysis of the hydrolysate of beet molasses showed the need to adjust some elements to balance with the physiological needs of the studied bacterial strain *C. necator* B-10646. To reduce the excess content of sugars and nitrogen, and remove the deficiency of phosphorus, which negatively affects the biomass concentration and PHA content, controlled modes of replenishment of the batch culture of bacteria with phosphorus and glucose were proposed and implemented, thereby providing high production rates for the process. Depending on the ratio of sugars introduced into the bacterial culture due to molasses hydrolysate and glucose additives, bacterial biomass concentrations were obtained from 20–25 to 80–85 g/L with a polymer content of up to 80%. A P(3HB-*co*-3HV) copolymer with minor inclusions of 3-hydroxyvlaerate monomers was the polymer synthesized on hydrolysates of molasses containing trace amounts of propionate and valerate. The introduction of precursor substrates into the culture of bacteria ensures the synthesis of copolymers of various compositions with reduced values of the degree of crystallinity, containing monomers of 3HV, 4HB, or 3HHx from 5–8 to 12–16 mol.%.

The studies have shown the possibility of productively synthesizing degradable polyhydroxyalkanoates from sugar beet molasses, a complex sugar-containing substrate, which is a large-tonnage waste of the sugar industry.

## Figures and Tables

**Table 1:** Indicators of sucrose hydrolysis and the composition of hexoses formed.

Molasses Hydrolysis Method	Glucose, g/L	Fructose, g/L	Hydrolysis Time, h	Degree of Hydrolysis, %
Acid	224.7	73.7	3	51.0
Enzymatic	258.9	261.4	24	88.9

**Table 2:** Chemical composition of sugar beet molasses and the resulting hydrolysate.

**Type of Molasses**	**Macroelements, g/L**						
	**Na**	**K**	**P**	**Fe**	**Mg**	**S**	**N Total**
Molasses initial	17.295	44.324	0.107	0.077	0.216	1.563	25.825
Molasses hydrolysate clarified	17.126	44.324	0.107	0.046	0.216	1.563	25.825
**Type of Molasses**	**Trace Elements, mg/L**						
	**B**	**Co**	**Cu**	**Mn**	**Zn**	**Mo**	**Ni**
Molasses initial	6.118	1.050	0.518	15.862	11.424	0.420	4.074
Molasses hydrolysate clarified	2.856	0.756	0.518	11.704	11.424	0.252	2.758

**Table 3:** Properties of PHAs synthesized *Cupriavidus necator* B-10646 from sugar beet molasses hydrolysate.

PHAs Composition, mol.%	Number Average Molecular Weight, M_n_, kDa	Weigh Average Molecular Weight, M_w_, kDa	Polydispersity, Ð	Degree of Crystallinity, C_x_, %	Melting Point, T_melt_, °C	Thermal Degradation Temperature, T_degr_, °C
C-substrate–molasses hydrolysate						
2 L Flask P(3HB-*co*-0.4mol.%3HV)	410	820	2.6	82	173.2	287.4
8 L Fermenter P(3HB-*co*-0.5mol.%3HV)	356	996	2.8	78	169.0 176.1	276.2
8 L Fermenter P(3HB-*co*-0.3mol.%3HV)	506	1214	2.4	76	169.4 179.7	276.0
C-substrate–molasses hydrolysate + precursors						
P(3HB-*co*-4HB) *						
P(3HB-*co*-6.5mol.%4HB)	144	461	3.2	45	152.0 163.8	286.3
P(3HB-*co*-12.0mol.%4HB)	200	600	3.0	38	153.2 167.8	288.0
P(3HB-*co*-3HV)						
P(3HB-*co*-8.0mol.%3HV)	212	678	3.2	67	163.5 172.2	283.5
P(3HB-*co*-16.5mol.%3HV)	200	740	3.7	55	167.4 169.0	287.0
P(3HB-*co*-3HHx) **						
P(3HB-*co*-5.6mol.%3HHx)	210	756	3.6	70	161.1 169.4	283.2
P(3HB-*co*-10.3mol.%3HHx)	180	738	4.1	68	165.4 167.8	270.0

Note: Minor inclusions of 3HV monomers are not shown, less than 0.5 mol.% in copolymers. * P(3HB-co-4HB) and ** P(3HB-co-3HHx).

## Data Availability

All data is available in the paper.

## Supplementary Materials

The following supporting information can be downloaded at: https://www.mdpi.com/article/10.3390/bioengineering9040154/s1, Figure S1: Results of thermal analysis of PHAs synthesized by *Cupriavidus necator* B-10646 from molasses hydrolysate.
