# Supplementary material for: Sugar Beet Molasses as a Potential C-Substrate for PHA Production by Cupriavidus necator
Source: Bioengineering (Basel). 2022 Apr 4;9(4):154. doi: 10.3390/bioengineering9040154 (PMC9031461; doi:10.3390/bioengineering9040154)
Supplement: Supplementary file 1 [file bioengineering-09-00154-s001.zip › bioengineering-1642396-supplementary.pdf]

Supplementary Materials: Sugar beet molasses as a potential C-substrate for PHA production by a wild strain Cupriavidus necator

Evgeniy G. Kiselev, Aleksey V. Demidenko, Natalia O. Zhila , Ekaterina I. Shishatskaya and Tatiana G. Volova

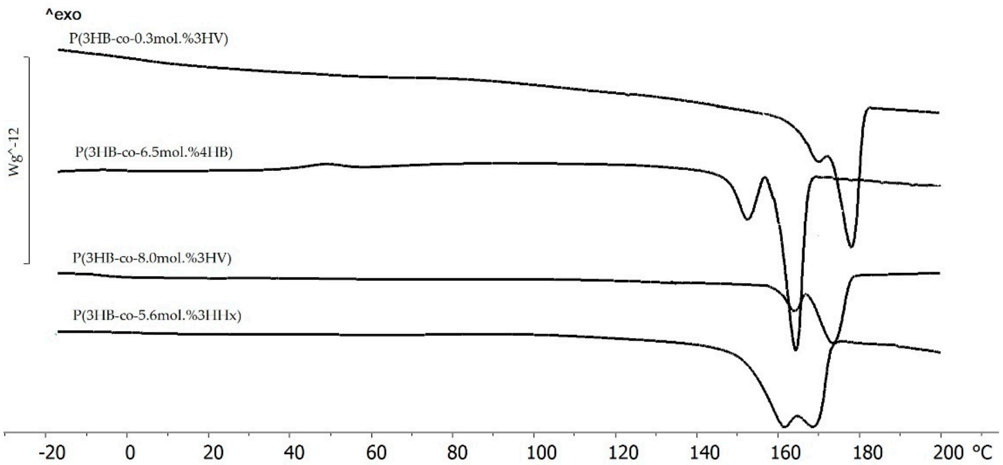

Figure S1. Results of thermal analysis of PHAs synthesized by *Cupriavidus necator* B-10646 from molasses hydrolysate.
